# Predictors of academic engagement of high school students: academic socialization and motivational beliefs

**DOI:** 10.3389/fpsyg.2024.1347163

**Published:** 2024-07-25

**Authors:** Getachew Tassew Woreta

**Affiliations:** Department of Psychology, Wollo University, Dessie, Ethiopia

**Keywords:** academic engagement, academic socialization, motivational beliefs, self-efficacy, outcome expectations, high school, peer academic socialization, parental academic socialization

## Abstract

The potential of academic engagement to enhance academic outcomes and well-being has been empirically supported, yet studies addressing its predictors are too limited. Hence, the current study collected self-report cross-sectional data from randomly selected 614 (male = 323) high school students in Ethiopia and examined the relations of academic socialization (parental and peer), self-efficacy, and outcome expectations in explaining variance in academic engagement, guided by an integrative model of engagement. Structural equation modeling with the maximum likelihood method indicated that (a) the hypothesized model fit the data well, (b) direct paths from parental and peer academic socialization to self-efficacy, outcome expectations, and academic engagement were positive and significant, (c) the relationships of the constructs in the model explained a significant portion of the variance in academic engagement, and (d) self-efficacy and outcome expectations significantly and positively but partially mediated the pathway from academic socialization to academic engagement. The findings’ implications for boosting student academic engagement were forwarded.

## Introduction

Researchers in education and educational psychology have given due attention to student engagement scholarship because it has been recognized mainly for the roles it plays in enhancing students’ learning (Skinner, 2016), persistence in education (Fredricks et al., 2004), academic achievement (Zumbrunn et al., 2014), school completion (Fall and Roberts, 2012), and better well-being (Wang et al., 2015; Moreira et al., 2019). Despite these and other well-noticed and duly acknowledged contributions that student engagement has, little has been done about what factors contribute to its development. This condition actually calls for a study aimed at identifying the potential antecedents of engagement and suggesting possible evidence-based interventions. Furthermore, the construct is highly responsive to changes in the environment (Wang et al., 2016) and malleable. Therefore, the purpose of the present study was to examine some contextual and personal antecedents of academic engagement among Ethiopian high school students. In this pursuit, an academic engagement model, which has been formulated based on a development-in-sociocultural context perspective (Wang et al., 2019, 2020) and prior empirical evidence, was tested with data collected from Ethiopian high school students.

### Positive outcomes of academic engagement

Literature on student engagement suggests that a substantial variation exists in how engagement has been defined and conceptualized and this has resulted in (a) the inclusion of multiple variables associated with student success in school, such as students’ school-related conduct (Fredricks et al., 2004; Wang et al., 2019), school belongingness (Finn and Zimmer, 2012), self-regulation (Greene, 2015), and future aspirations and goals (Appleton et al., 2006), (b) measuring student engagement via different tools, (c) inclusion of two, three, or four dimensions (Fredricks et al., 2004; Reeve and Tseng, 2011; Wang et al., 2016), (d) confusion about the facilitators and the indicators of engagement (Lam et al., 2014), and (e) using the same items to measure different aspects of engagement (Eccles and Wang, 2012) which finally caused difficulty in comparing the reported findings.

Nevertheless, despite the discrepancies in conceptualization and measurement of student engagement, studies provided adequate empirical support for the positive effect of engagement on students’ academic performance at all educational levels (Wang and Holcombe, 2010; Dotterer and Lowe, 2011; Li and Lerner, 2011; Finn and Zimmer, 2012; Reeve and Lee, 2014; Dogan, 2017; Fung et al., 2018). The existing empirical works have witnessed the roles of student engagement in enhancing academic success and school completion (e.g., Archambault et al., 2009) and decreasing the risk of school dropout and delinquency (Fall and Roberts, 2012; Henry et al., 2012; Wang and Peck, 2013; Wang and Fredricks, 2014). Student engagement also has long-term effects on emotional well-being and adjustment. The research found that student engagement was positively associated with adjustment (Simons-Morton and Crump, 2003), use of practical coping skills (Reschly et al., 2008), less depressive symptoms (Li and Lerner, 2011), and well-being (Reschly et al., 2008; Salmela-Aro et al., 2009; Cadime et al., 2016; Boulton et al., 2019). Students with positive pathways of engagement are less likely to show problem behaviors (i.e., less likely to be involved in delinquency, serious offenses, and problem substance use) during adolescence and early adulthood (Hirschfield and Gasper, 2011; Li and Lerner, 2011; Henry et al., 2012). Findings from data spanning 40 years of life, taking into account many individual difference variables, also showed that adolescent school engagement had a positive impact on adult educational and employment outcomes (Symonds et al., 2023).

Given that academic engagement plays multifaceted roles in the life of students and it is malleable (Bresó et al., 2011; Wang and Eccles, 2012), more effort has to be invested in identifying what factors positively contribute to its development. In this regard, Alrashidi et al. (2016) noted that “it is important for researchers and educators to consider factors that might help heighten and foster students’ engagement in school and academic-related activities which, eventually, enhance students’ performance outcomes” (p. 47). Although few researchers have recently begun to respond to this call (e.g., Jang et al., 2010; Johnson and Sinatra, 2013; Zhen et al., 2018), more research focusing on what factors shape student engagement in the learning process needs to be conducted to identify appropriate areas of interventions promoting students’ academic engagement. The facilitators of student engagement can be broadly grouped into self-system (personal) and social system (contextual), although they can also be further separated into other classes of variables (Wang and Holcombe, 2010; Bresó et al., 2011; Wang and Eccles, 2012, 2013; Phan and Ngu, 2014; Reeve and Lee, 2014). In this study, academic socialization (parent & peer) represents contextual factors, while self-efficacy and outcome expectations belong to the class of personal factors.

### Theoretical framework

**Figure 1:**
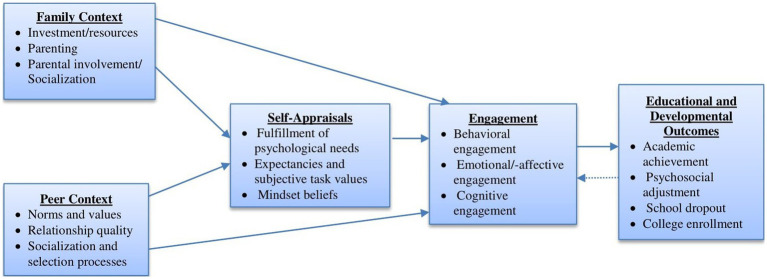
Some aspects of development-in-sociocultural context model of engagement (Wang et al., 2019).

The structural relationships among the constructs included in the study were guided by an integrative theoretical model of engagement, also called a development-in-sociocultural context perspective (Wang et al., 2019, 2020). An integrative theoretical model of engagement was developed in an attempt to respond to scholars’ accentuated need for “a synthetic, coherent framework that simultaneously integrates extant literature and clarifies the conceptualization of engagement, identifies its facilitators and consequences, and proffers a theoretical model that elaborates on how engagement functions” (Wang et al., 2019, p. 1087). In response to such need for a theoretically sound, integrative model among the scholarly community, Wang and his colleagues synthesized extant research and integrated relevant concepts from self-system theory (Skinner et al., 2009), expectancy-value theory (Eccles, 2009), and mindset theory (Dweck, 2006) to illuminate the motivational processes underlying engagement. This recent and integrative theoretical perspective accounts for how student academic engagement develops over time and provides a theoretical framework for organizing the predictors and outcomes of student engagement, depicting the structural relations between social context, ‘self,’ engagement, and outcomes (Figure 1 depicts aspects of development-in-sociocultural context model related to the present study). As to the structural relationships between contexts, self, and engagement, the integrative theoretical perspective posits multiple direct and indirect (mediated by self-related variables) causal pathways from social contexts to academic engagement. The integrative theoretical model for children’s engagement in learning is a complex developmental system that incorporates many categories of contextual and personal factors. The present study, however, examined only the structural relations of four constructs (parental academic socialization, peer academic socialization, academic self-efficacy, and educational outcome expectations) with students’ academic engagement. For example, the model posits that motivational beliefs (e.g., self-efficacy and outcome expectancy) partially mediate the causal path from social context to student engagement. According to the integrative model, students’ socialization experiences in family and peer contexts determine their self-efficacy and outcome expectations; in turn, the level of academic self-efficacy and outcome expectations influence the level of students’ academic engagement. That is, the academic socialization that children experience at home and in peer contexts over time accumulates to shape their academic self-efficacy and outcome expectations for learning, which in turn influences their engagement in learning. While the development-in-sociocultural context model suggests causal links from left to right (social context → self → engagement → outcomes), it acknowledges the possibility of intricate feedback cycles or bidirectional processes in certain instances. For example, high-quality academic engagement could reinforce children’s emerging self-appraisals and competencies and determine the level of support from adults and the peer selection process. However, variable specific evidence is needed, instead of broad categories of constructs, to consider specific paths in opposite directions.

### Academic socialization and student academic engagement

Parental involvement is vital for their children’s school engagement and success (Melby et al., 2008; Murray, 2009; Fan and Williams, 2010; Fan et al., 2012; Wilder, 2014), although the impact of each form of involvement may vary depending on the developmental stage. Among other states of parental involvement (e.g., attending school events, helping with homework), parental academic socialization (socializing messages) like relating education to future success (Wang et al., 2014), is a vital aspect of the family context for understanding how parents influence their children’s schooling and educational success during the period of adolescence (Hill and Tyson, 2009; Wang et al., 2014; Bæck, 2017).

Nowadays, academic-focused messages that parents convey to their children are considered an important aspect of parental involvement practices in influencing children’s academic outcomes (Hoover-Dempsey and Sandler, 1997; Bempechat et al., 1999; Suizzo and Soon, 2006; Hill and Tyson, 2009) and such form of parental involvement represents parental academic socialization. Academic socialization is a verbal form of parental involvement that denotes academic-related messages exchanged between the parents and adolescent students rather than direct behavioral (e.g., social control & monitoring) or instrumental involvements (e.g., assisting with schoolwork, supplying educational resources). The most common academic messages from parents, representing academic socialization, relate to the value of education, the importance of effort, the pressure to meet parental expectations, and the shame of not meeting academic standards (Bempechat et al., 1999; Suizzo and Soon, 2006). In this study, parental academic socialization included messages aimed at socializing children about the importance of academic effort, the values of education or the value of educational success, and educational expectations. Effort socialization represents those parental messages conveying the value of being effortful and hard-working, as well as associating failure in academic performance and career development with a lack of effort. It denotes inspiring the need to put forth the best academic effort for better academic and career development. Evidence indicates that effort socialization was related to motivational outcomes such as locus of control and classroom engagement (Suizzo et al., 2012, 2016). Parents also socialize their children by conveying their expectations for educational attainment. Educational expectations refer to the anticipation of accomplishment at school or university (Mello, 2008). Parents’ expectations about their children’s future achievement can be expressed in course grades, the highest level of schooling attained, or college/university attendance (Goldenberg et al., 2001; Glick and White, 2004). Research shows that children with high expectations from their parents do better academically, perform better on standardized tests, and stay in school longer than children with low expectations from their parents (Davis-Kean, 2005; Pearce, 2006; Vartanian et al., 2007). Meta-analyses studies also indicated that parental educational expectations are the strongest family-level predictor of student academic outcomes relative to other parental beliefs and behaviors (Jeynes, 2005, 2007). Therefore, educational expectations, values of education, and the importance of effort that parents convey to their children define parental academic socialization and are assumed to influence students’ academic engagement.

During the adolescence period, peer context also becomes the most crucial aspect of the social system in socializing academic behaviors of adolescent students positively (Altermatt and Pomerantz, 2003; Kindermann, 2007; Rodkin and Ryan, 2012). Peer socialization is the process by which individuals learn many things from their peers, particularly from friends with whom they spend much of their time (Ryan, 2000; Kindermann and Gest, 2009). The context of the peer group determines the degree of an individual student’s engagement in academic tasks, either by promoting or discouraging academic attitudes, values, and behaviors (Wang et al., 2018). Hanging out with peers who are academically motivated and committed increases one’s motivation and commitment over time, although the opposite is also true. Evidence from longitudinal data indicated that friends and peers do socialize with each other over time in their academic motivation, engagement, and achievement (Ryan, 2001; Altermatt and Pomerantz, 2003; Kindermann, 2007).

Observation of other peer members changes one’s cognition, behavior, or emotions (Ryan et al., 2019). The behaviors, beliefs, attitudes, values, and other characteristics reflected and demonstrated by the peers can introduce a student to new behaviors, thoughts, perspectives, and feelings either through the process of modeling, the exchange of persuasive messages, or social reinforcement (encouragement or discouragement by peers). Research has indicated that friends are similar in their effortful behavior toward their schoolwork, interest, and enjoyment in their schoolwork (Ryan, 2001; Shin and Ryan, 2014), and preference for challenges (Altermatt and Pomerantz, 2003). These similarities among friends suggest how peers matter in socializing students’ behavioral, affective, and cognitive engagement (Ryan et al., 2019). Adolescent peers interact regularly and spend more time together; hence, they have more opportunities to model and influence each other’s academic behaviors (Wentzel, 2009). However, the attention given to peers matters for students’ academic engagement is not as much as it deserves (Steenberghs et al., 2021). Specifically, peer influence through academic socialization did not receive the attention of the researchers in student engagement research.

Therefore, this study approached peer influence from an academic socialization perspective that includes three components: peers’ educational aspirations, peers’ efforts, and peers’ academic norms. The extent to which students in a peer group exhibit positive academic behaviors (e.g., completing homework, attending class, valuing high grades, etc.) represents the academic norms of peers (McCormick and Cappella, 2014). The academic norms prevailing in the peer group are assumed to be imperative in determining high school student academic engagement. Friends and classmates were found to contribute to the development of individual student’s behavioral and emotional engagement and disengagement (Steenberghs et al., 2021). Wang et al. (2018) also, using the peer nomination approach, found that over time students became more similar to the peers they nominated in their engagement. It is also important to note that in the early years of high school, students begin to think about their future education and careers; hence, they often discuss their views and aspirations with their peers (Eccles et al., 2004; Kiuru et al., 2007). Therefore, the aspirations of other students in the peer groups may be another way in which peers socialize students’ educational aspirations and academic engagement. Adolescents tend to demonstrate similar levels of school-related adjustment to others in their peer group (Ryan, 2001; Chen et al., 2003). In a collectivist society like Ethiopia, the likelihood of being influenced by the perceived views of others in the peer group is believed to be high. Accordingly, the construct of peer academic socialization included peers’ educational aspirations based on findings from other settings and the culture of current participants.

In this study, the peer/friendship group consists of individuals from different schools and classrooms but from the same grade level because a student in a collective society could have friends who are not in the same school but in the same residential area. In the context of Ethiopia, beyond the time spent in the classroom, students walk a long distance in a group from home to school or vis-versa which allows them to exchange more information and influence each other’s behavior. In addition to walking a long distance in a group, students from different schools could meet regularly at home or in areas around their homes to do schoolwork and study for tests together, allowing for more discussions and information exchange about school lessons. As a result, feelings, behaviors, and thoughts regarding learning and school might be shared repeatedly among peer members and influence what students feel, think, and do in school. In Ethiopia, the probability of a student’s academic motivation and behavior being determined by a peer group is higher than the peer context of the Western world, which is dominantly individualistic in life. However, the attention given to the effect of peers on students’ academic matters is not as it deserves, and almost none in the context of Ethiopian high school students.

### Motivational beliefs and student academic engagement

In addition to contextual factors, personal or individual-level factors play a vital role in shaping student engagement in academic affairs. Outcome expectations and self-efficacy are the most important cognitive-motivational factors that positively influence learning engagement, according to social cognitive theory (Bandura, 1997). Academic self-efficacy refers to belief in one’s ability to accomplish academic tasks (Bandura, 1997). As self-efficacy beliefs importantly determine one’s effort expenditure, emotional responses, and sense of persistence in carrying out challenging academic tasks, students who have high academic self-efficacy beliefs tend to show greater academic engagement (Martin and Rimm-Kaufman, 2015; Rimm-Kaufman et al., 2015; Liu et al., 2018; Granziera and Perera, 2019; Navarro et al., 2019). It was found that higher self-efficacy beliefs were associated with greater engagement among fifth graders (Rimm-Kaufman et al., 2015) and university students (Wilson et al., 2015). However, self-efficacy alone does not motivate students to engage in academic activities (Wang and Degol, 2014) because outcome expectations for being engaged in the tasks also determine students’ initiation and the decision to engage in the tasks. Outcome expectations uniquely account for motivated actions over and above self-efficacy beliefs (Fouad and Guillen, 2006). Outcome expectations represent the probable outcomes an individual expects for engaging in a particular course of action. They stand for the question, “If I do this, what are the consequences?” (Lent et al., 1994, p. 83). Educational outcome expectations, specifically, denote the probable outcomes a student expects for engaging in academic or educational activities. The role of outcome expectations has been well recognized in career development theories and research, especially in predicting career outcomes, such as interests, goals, persistence, and performance (Lent et al., 1994; Byars-Winston et al., 2010; Lent et al., 2013; Flores et al., 2014; Lent et al., 2016), though less attention is given to be incorporated into student engagement research. If an individual expects more positive outcomes for performing a particular task, the likelihood of being engaged in that task is greater (Bandura, 1997; Fouad and Guillen, 2006). Task values are positively related to engagement (Fan and Williams, 2010; Fan, 2011; Wang and Eccles, 2013) and the intention to continue studying at school or beyond (Fan and Wolters, 2014). Despite a shortage of studies addressing the relationship between outcome expectations and indices of academic engagement, Navarro et al. (2019) reported a positive and unique contribution of engineering outcome expectations to engineering academic engagement based on data collected from undergraduate engineering students. Hence, in the present study, beliefs in one’s academic ability and the expected outcomes are posited to influence the degree of students’ academic engagement. Self-efficacy and outcome expectations are assumed to be the functions of equivalent learning experiences or contexts, so incorporating them simultaneously in a model that incorporates social context as their antecedents and academic engagement as outcomes seems to be sensible.

### The current study

**Figure 2:**
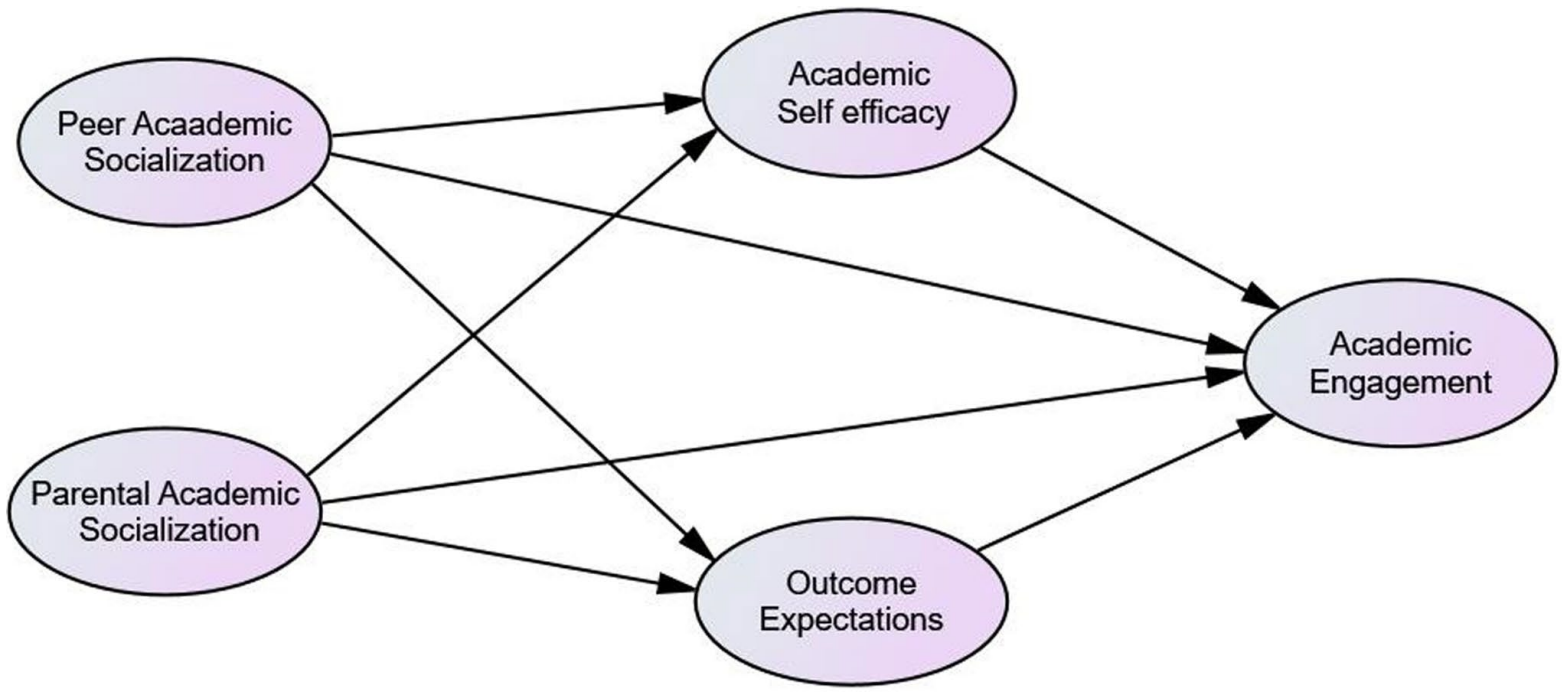
Proposed structural model of academic socialization, motivational beliefs and academic engagement.

Although the role of student engagement in fostering learning outcomes has a substantial amount of empirical support regardless of the level of schooling, studies that examined what factors and how they determine the development of high school students’ academic engagement were too limited. More research is needed to understand the dynamics through which contextual and personal factors shape student engagement and to improve the level and quality of students’ academic engagement and, in turn, to increase their academic success. Therefore, the purpose of the current study was to examine the contextual and personal antecedents that could contribute a substantial amount of variance in the academic engagement of high school students. Based on the integrative theoretical perspective on engagement (Wang et al., 2019, 2020) and prior empirical works, academic socialization experiences from peers and parents, academic self-efficacy, and educational outcome expectations were assumed to explain a substantial amount of variance in high school students’ academic engagement. Hence, this study examined the relations of parental and peer academic socialization with student academic engagement, treating self-efficacy and outcome expectations as mediators to indicate how parents and peers shape students’ academic engagement. The model proposed for the present study (Figure 2) depicts the direct links posited between academic socialization and academic engagement as well as indirect pathways (via academic self-efficacy and outcome expectations) through which the socialization process shapes student engagement in learning. The primary hypothesis is that if students experience positive parental and peer academic socialization, feel efficacious in their academic capability, and expect that their academic pursuit will result in significant outcomes, they will demonstrate greater academic engagement. For instance, parental messages that stress the need to exert academic efforts, convey positive values of education, and communicate high expectations to their children would have both direct and indirect links (via academic self-efficacy and educational outcome expectations) to the academic engagement of high school students.

The constructs included in the model and the presumed structural relations with academic engagement were found to be considerable because no studies have investigated these antecedents simultaneously within one conceptual framework using SEM. Specifically, the proposed model was not addressed by prior studies. Moreover, in this study: (1) the conceptualization of academic socialization and associated indices, which carefully considered the existing literature so far, was different from previous studies, and (2) the way academic engagement was defined and measured is unique because it was guided by the most recently proposed perspective on student engagement (Wong and Liem, 2022). The current study defined academic engagement as “students’ psychological state of activity that affords them to feel activated, exert effort, and be absorbed during learning activities” (Wong and Liem, 2022, p. 120), with emotional, behavioral, and cognitive dimensions. Within this conceptualization, emotional engagement represents the level of activation that students experience during learning activities and is indicated by positive feelings, such as vigor, interest, enjoyment, and alertness. Feelings of attachment to the school and its community, unlike most of the prior studies, were not included in emotional engagement because they represent school engagement rather than learning engagement. Feelings of being connected to school (reflected by school attachment, belonging, bonding, and identification) and feelings of connection, closeness, and supportive relationships with teachers and classmates are not indicators of but rather potential antecedents of engagement (Skinner, 2016). Behavioral engagement represents the extent of students’ deliberate effort they exert during learning activities and can be measured by a self-report tool that includes items tapping the students’ effort and persistence during learning activities (Wong and Liem, 2022). Cognitive engagement, the third dimension, denotes the level of student absorption during learning activities, being marked by (a) high-level concentration and task-relevant thoughts and (b) a decline in awareness of irrelevant external events (Wong and Liem, 2022). Scholars (e.g., Ben-Eliyahu et al., 2018; Wong and Liem, 2022) note that cognitive engagement represents the general cognitive processes like absorption instead of the specific learning strategies that students use during learning. Accordingly, Wong and Liem (2022) suggest that researchers can use self-reports of absorption during learning activities (Salmela-Aro and Upadaya, 2012) to assess student cognitive engagement. Unlike most of the prior studies, variables such as attendance, positive conduct, connection with the school and its community, and use of self-regulated learning strategies were not included in the present conceptualization of academic engagement because, most perhaps, they are antecedents or prerequisites of engagement instead of indicators.

The uniqueness of this study is not only in the simultaneous inclusion of the stated constructs in a single model, the order of placement within the model, and the way some of the constructs were conceptualized and measured but also in the context in which the study was conducted. It may be worth assuming that the contributions of social and psychological variables could differ across populations. Some studies that attempted to examine the predictors of student engagement were in the Western culture, and the extent to which the results can be applicable to non-Western contexts, specifically in the context of Ethiopia, is unseen.

It is important to note that there are many variations between the Western and Ethiopian contexts. The context in Ethiopia is different from the Western context, mainly in terms of culture and socio-economic factors. Western countries endorse individualism and individuals seek to maintain personal autonomy by attending to the self, while Ethiopia advocates collectivism in which the relatedness of individuals to each other and community values are more valued (Markus and Kitayama, 1991). This variation, as noted by Markus and Kitayama, has important effects on cognition, emotion, and motivation. In terms of the level of economic development and technological advancement, Western society is generally higher than that of Ethiopia. Ethiopia is still considered a developing country with a less diversified economy. Such difference also results in variations in the lifestyle, education, and opportunities available to people in these contexts. However, many scientific theories and hypotheses have been developed and tested primarily in Western contexts. Henrich et al. (2010) highlighted the fact that a significant portion of the psychological literature is built on studies conducted within Western, Educated, Industrialized, Rich, and Democratic (WEIRD) societies. This raises questions of generalizability to other cultures and societies around the world, highlighting the need for greater diversity and inclusivity in psychological research. Therefore, testing hypotheses generated based on Western contexts in Ethiopia can be plausible because cultural and socioeconomic contexts can greatly affect the applicability of these hypotheses in other parts of the world, particularly in developing countries like Ethiopia. Ethiopia may provide a context that is different enough from Western contexts to test the extent to which Western-based theories and hypotheses can be generalized across cultures and to establish the external validity of findings from Western populations. Evidence indicated that (e.g., Klassen, 2004; Ahn et al., 2016), students from individualistic and collectivistic cultural backgrounds evaluated and interpreted socially conveyed sources of motivational beliefs (e.g., vicarious experience and social persuasion) differently in which for students with collectivistic cultural backgrounds socially conveyed messages were the most important sources for academic self-efficacy formation. Given that Ethiopia’s cultural, social, and economic contexts, as well as its education system significantly differ from those of other countries where theoretical hypotheses are generated and previous studies have been conducted, and considering the substantial influence of cultural contexts, the motive behind the current study seems convincing.

Findings from this study would (a) strengthen the understanding of what factors positively shape student academic engagement across cultures. (b) provide empirical support for the cross-cultural validity of a development-in-sociocultural context theoretical framework, (c) suggest interventions to be initiated and implemented to improve the academic performance of students through augmenting academic engagement, and (d) in general help to expand our understanding of human behavior and psychology beyond the Western contexts. This study has particular importance in the context of Ethiopia, where high school students are becoming lower and lower in their academic motivation, engagement, and achievement to a great extent. For example, among grade 12 students who took Ethiopia’s 2022 national school-leaving examination, only 3.3% of them were able to score 50% and above to qualify for tertiary education.

## Method

### Participants

The participants were 614 (323 males and 291 females) randomly selected high school students in grades 9 (*n* = 329) and 10 (*n* = 285). They were from six randomly chosen public high schools located in six different districts of South Wollo Administrative Zone, Amhara Regional State, Ethiopia. From the six schools, one to three classes of each target grade were selected randomly, and finally, each participant was chosen with a simple random sampling technique. The number of participants across schools ranges from 60 to 145, based on the total number of students in each sample school. The sample had a mean age of 16.1 years (SD = 0.66), ranging from 15 to 17 years of age. All the participants attend regular high school education programs in public schools. Ethnically, participants were Amhara except that 13 students identified as other ethnicities and three students did not indicate.

### Procedures

Following receipt of ethical approval from the ethics committee of the Institute of Teachers Education and Behavioural Science, Wollo University, the school principals and teaching staff at each school were approached, and their permission to collect the data was obtained. School teachers administered the questionnaire to participants during the school days, with no time limits and close supervision of the researcher. Students provided their informed consent (dealing with their parents) by singing on the consent forms prepared for such purpose. However, participants were not requested to write a name or other personal identifying variables to ensure the anonymity of data.

### Measures

#### Academic engagement

The academic engagement measure included 16 items. For behavioral and emotional dimensions, the engagement subscales of the Engagement Versus Disaffection with Learning measure (Skinner et al., 2009) were used except for the two items added to the behavioral subscale from Wang et al. (2016). The behavioral engagement scale comprised six items tapping students’ effort and persistence while participating in learning activities. The emotional engagement subscale included five items tapping positive emotions during learning activities. The cognitive engagement was assessed by five items, which included the three-item absorption subscale of the schoolwork engagement inventory (Salmela-Aro and Upadaya, 2012) and two items from the cognitive engagement scale designed by Ben-Eliyahu et al. (2018). This was done to be consistent with the way cognitive engagement has been conceptualized and the recommendation forwarded by Wong and Liem (2022). Moreover, items addressing learning engagement at home were included. Sample items include: “I try hard to do well in school” (behavioral engagement), “Class is fun for me” (emotional engagement), and “Time flies when I am studying” (cognitive engagement). Participants rated the items using a 5-point Likert-type scale, with the options of ‘not at all true for me’ (1) to ‘very true for me’ (5). Previous studies have disclosed adequate reliability estimates for the original items of each dimension (Skinner et al., 2008; Salmela-Aro and Upadaya, 2012; Xiong et al., 2021). The present data disclosed acceptable Cronbach’s alpha values (0.92 for behavioral, 0.89 for emotional, 0.90 for cognitive, and 0.89 for the full engagement scale). Confirmatory factor analysis (CFA) was conducted to assess each item’s factor loading and factor structure of the constructs included in this study. Within this paper, model fit was evaluated using five indices: the relative chi-square test (ꭓ^2^/df), the Tucker–Lewis index (TLI), the comparative fit index (CFI), the root mean square error of approximation (RMSEA), and the standardized root-mean-square residual (SRMR). Because of much controversy on appropriateness and interpretation of the model-fit criteria, Schumacker and Lomax (2016) recommend reporting more than one model-fit index stating that if “a majority of the fit indices on your list indicate an acceptable model, then your theoretical model is supported by the data” (p. 119). As the chi-square statistic (χ^2^) is affected by the sample size, it mostly rejects the model when the sample size increases (if *N* > 200) (Bentler and Bonett, 1980; Schumacker and Lomax, 2016). As a result, a relative chi-square statistic (χ^2^/df) was used instead of it in this study (Wheaton et al., 1977). If CFI and TLI ≥ 0.95, and RMSEA and SRMR ≤0.06 (Hu and Bentler, 1999), a model adequately fits the data. A relative chi-square test with a value below 3 is considered an acceptable fit (Kline, 2011). For the academic engagement measure, CFA suggested that the three-factor model fit the data well (ꭓ^2^ /df = 3.2, CFI = 0.96, TLI = 0.95, SRMR = 0.036, RMSEA = 0.06, 90% CI = 0.053, 0.067). Factor loadings reached statistical significance and their standardized estimates ranged from 0.749 to 0.85.

#### Parental academic socialization

The parental academic socialization (PAS) scale has 17 items and assesses parents’ messages of effort socialization (4 items), educational expectations (5 items), and value of education (8 items). Participants indicated the extent to which they have been experiencing such parental messages from 1 (*not at all*) to 5 (*always*). Effort socialization items were from the effort subscale of the Educational Socialization Scale (Bempechat et al., 1999). A sample item from the effort subscale includes, “My parents say you can get smarter and smarter as long as you try hard.” For parental educational expectations, five items were adapted from the perceived parental academic support scale (Chen, 2005; Cross et al., 2019). This scale included items such as “I feel pressured by my parents to do well in school.” The value or importance of education sub-scale consisted of the five-item Benefits of Education subscale of the Economic Benefits and Limitations of Education scale (Murdock et al., 2000) and three items created for the present study to assess the non-economic value of education. A sample item includes: “My parents say if I do well in school, I will get a good job,” Higher scores in each dimension of parental academic socialization reflect a greater frequency of the PAS messages. Previous studies have disclosed adequate reliability estimates for the original items of each dimension (Murdock et al., 2000; Mroczkowski and Sanchez, 2015; Cross et al., 2019). In the present study, the data disclosed Cronbach’s alpha coefficient of 0.86, 0.92, 0.84, and 0.89 for effort, the value of education, educational expectations, and full scale, respectively. CFA also suggested that the three-factor PAS model fit the data well (ꭓ^2^ /df = 2.85, CFI = 0.96, TLI = 0.95, SRMR = 0.04, RMSEA = 0.055, 90% CI = 0.048, 0.062). Factor loadings reached statistical significance and their standardized estimates ranged from 0.72 to 0.82.

#### Peer academic socialization

Participants were asked to list a group of friends with whom they spend more time and do many things together, without limit to the number of friends to be listed. They were informed that members of the friendship group they list are limited to their grade level, but they can be from other classrooms and schools. What matters in the selection process is the time spent together and feelings of connectedness to do things together and exchange information. Participants were reminded that the members of this group were referred to as “your friends or friendship group” throughout the items included to assess the context of their peer group. The purpose of the list of friends is to make a focused and pertinent judgment about the context of the peer group.

The measure of peer academic socialization consisted of 16 items and included three subscales. The effort socialization subscale has four items tapping the peer group’s stress on effort and was adapted from the effort subscale of the Educational Socialization Scale (Bempechat et al., 1999). A sample is, “My friends say we could do better in school if we worked harder.” Measures addressing peers’ academic norms and educational aspirations were adapted from the Peers’ Academic Support and Aspirations Scale (Murdock, 1999; Murdock et al., 2000). With regard to assessing peer academic norms, each participant was presented with the seven-item peers’ academic norm scale and asked to rate to what extent most of the members of his/her friendship group had the potential to do a variety of academic tasks and demonstrate positive academic behaviors relevant to high school education. A sample item used to assess the academic norms of the peer group includes: “Most of my friends try to do well in school.” Peers’ educational aspirations had five items to assess students’ perceptions that their friends would complete high school and continue with their education (peers’ aspirations for their academic future). This scale included items such as “Most of my friends plan to go to college/university.” The current study revealed good internal consistency for the peers’ effort subscale (Cronbach’s alpha = 0.77), educational aspirations subscale (Cronbach’s alpha = 0.82), academic norm subscale (Cronbach’s alpha = 0.84), and full academic socialization scale (Cronbach’s alpha = 0.87). CFA also demonstrated an excellent model-data fit for three-factor structure of peer academic socialization scale (ꭓ^2^ /df = 2.1, CFI = 0.97, TLI = 0.96, SRMR = 0.038, RMSEA = 0.043, 90% CI = 0.035, 0.051). Factor loadings reached statistical significance and their standardized estimates ranged from 0.62 to 0.73.

#### Academic self-efficacy

Academic self-efficacy was assessed by the academic self-efficacy subscale of the self-efficacy questionnaire for children (SEQ-C; Muris, 2001), which has eight items to tap students’ feelings about their ability to be successful in school and demonstrate appropriate academic behaviors. The participants rated their feeling of efficacy using a four-Likert-type scale (1 = not at all confident to 4 = very confident). The scale included items like “How confident are you that you could study when there are other interesting things to do?” An SEQ-C drew greatly on the concept of self-efficacy, and it has been acknowledged in many of its features, such as it was developed with youth, is simple in terms of format, is domain-specific, and is fairly brief (Minter and Pritzker, 2017). The exploratory factor analysis result indicated that all items of the scale “hung together” to form a one-dimensional scale. CFA also supported the unidimensionalty of the measure (ꭓ^2^/df = 2.3, CFI = 0.99, TLI = 0.99, SRMR = 0.018, RMSEA = 0.046, 90% CI = 0.028, 0.064). Factor loadings reached statistical significance and their standardized estimates ranged from 0.75 to 0.79. The scale demonstrated a strong internal consistency in previous research (e.g., Landon et al., 2007; Suldo and Shaffer, 2007; Minter and Pritzker, 2017), which also appeared to be good for the current participants (Cronbach’s alpha = 0.92).

#### Educational outcome expectations

The outcome expectations measure consisted of 14 items taken from the College Outcome Expectations questionnaire (Flores et al., 2008). The items were adapted to the study population by replacing the stem: “A college education will...” with “secondary and post-secondary education will …” The original scale has 19 items, but for the present study, four items related to social affairs (i.e., leaving enough time for family and friends, making several friends, meeting new people, and causing problems in the family) and one item related to college courses, totally five items, were excluded as they are found not applicable for the present participants. Participants rated each item (e.g., “Secondary and post-secondary education will allow me to obtain a well-paying job”) on a 5-point scale ranging from 1 (strongly disagree) to 5 (strongly agree). High scores represent a high level of positive academic outcome expectations. The output of exploratory factor analysis suggested that all items of the scale “hung together” to form a one-dimensional scale. CFA also supported the unidimensionalty of the measure (ꭓ^2^/df = 3.2, CFI = 0.97, TLI = 0.96, SRMR = 0.028, RMSEA = 0.06, 90% CI = 0.051, 0.069). Factor loadings reached statistical significance and their standardized estimates ranged from 0.70 to 0.81. In the present study, Cronbach’s alpha value was 0.94.

## Results

### Preliminary analyses

During data screening, a closer examination of the data revealed that 21 cases were missing more than 20% of the items and were deleted from the data set (Schlomer et al., 2010; Navarro et al., 2019). The deletion has reduced the data set to 614. In testing the measurement model, three latent variables were represented by their corresponding subscales. Accordingly, parental academic socialization consisted of educational expectations, effort socialization, and value of education; peer academic socialization included peers’ educational aspiration, peers’ effort, and peers’ academic norms; and academic engagement comprised behavioral, emotional, and cognitive dimensions. Self-efficacy and outcome expectations were each indexed by three item parcels formed by random algorithm (Matsunaga, 2008). The number of items in the parcels of academic self-efficacy was from 2 to 3 (ASE1 includes items 1, 2, and 4, ASE2 includes items 3, 6, and 8, and ASE3 includes items 5 and 7), whereas, in outcome expectations, it was from four to five items (OE1 includes items 1, 2, 4, 5, and 7, OE2 includes items 3, 6, 8, 11, and 14, and OE3 includes items 9, 10, 12, and 13). In total, the model included 15 indicator-level variables. The purpose of the present study is to examine the relationships among constructs included in the proposed model rather than focusing on the relationships among individual items, therefore, “parceling is more strongly warranted”(Little et al., 2002, p. 169).

The data was checked for outliers and normality issues at indicator-level variables. The numerical standard deviates method, |*z*| < 3.0 (Kline, 2023), and the graphic techniques (e.g., box plots) did not display scores further away from the rest of the distribution; hence, there is no issue of univariate outliers in the dataset. Mahalonbis distance, *D*^2,^ also did not indicate evidence for multivariate outliers (i.e., a small value of *D*^2^ and lowest *p*-value, 0.001). The data was found to be univariate normal as all of the absolute values of the skewness (range: −0.43 to 0.345) and kurtosis (range: −0.783 to −0.218) indices were less than 2 and 7, respectively (Finney and DiStefano, 2013; Kline, 2023). Marida’s normalized estimate of multivariate kurtosis for the model was 4.45 (< 5), indicating no violation of multivariate normality (Byrne, 2016). None of the correlations among indicator variables after parceling (range 0.24 to 0.83) and among predictor latent constructs (range 0.35 to 0.66) surpassed 0.90, suggesting that multicollinearity was not a problem (Abu-Bader, 2010; Kline, 2023). Therefore, the final data set maintained responses from 614 participants.

The descriptive statistics and correlations among the study’s observable variables are presented in Table 1. The relations among the variables were significant. The standardized loadings of indicator variables on respective factors range from 0.76 to 0.92 (Figure 2).

**Table 1:** Descriptive statistics and correlations among the study’s observable variables.

	Variables	1	2	3	4	5	6	7	8	9	10	11	12	13	14	15
1	Peer educational aspiration	–														
2	Peer academic norm	0.58**	–													
3	Peer effort socialization	0.62**	0.61**	–												
4	Outcome expectations 1	0.40**	0.36**	0.43**	–											
5	Outcome expectations 2	0.37**	0.32**	0.38**	0.70**	–										
6	Outcome expectations 3	0.36**	0.35**	0.39**	0.64**	0.67**	–									
7	PAS – educational expectations	0.44**	0.43**	0.40**	0.45**	0.44**	0.41**	–								
8	PAS – effort	0.40**	0.42**	0.39**	0.40**	0.41**	0.45**	0.69**	–							
9	PAS – value of education	0.41**	0.42**	0.43**	0.50**	0.46**	0.42**	0.64**	0.61**	–						
10	Cognitive engagement	0.47**	0.44**	0.43**	0.47**	0.46**	0.45**	0.49**	0.51**	0.51**	–					
11	Behavioral engagement	0.48**	0.44**	0.45**	0.49**	0.47**	0.44**	0.53**	0.49**	0.48**	0.85**	–				
12	Emotional engagement	0.52**	0.44**	0.46**	0.44**	0.46**	0.42**	0.51**	0.50**	0.48**	0.78**	0.77**	–			
13	Academic self-efficacy 1	0.29**	0.37**	0.30**	0.22**	0.21**	0.16**	0.33**	0.34**	0.34**	0.36**	0.43**	0.38**	–		
14	Academic self-efficacy 2	0.32**	0.33**	0.30**	0.17**	0.18**	0.16**	0.35**	0.34**	0.29**	0.31**	0.33**	0.34**	0.59**	–	
15	Academic self-efficacy 3	0.28**	0.32**	0.26**	0.17**	0.15**	0.11**	0.28**	0.29**	0.29**	0.31**	0.33**	0.31**	0.64**	0.67**	–
	Mean	3.53	3.35	3.47	3.69	3.72	3.71	3.58	3.68	3.38	3.45	3.51	3.36	2.25	2.58	2.47
	Standard deviation	0.75	0.81	0.78	0.73	0.76	0.72	0.74	0.83	0.76	0.84	0.87	0.86	0.7	0.7	0.68
	Skewness	−0.41	−0.06	−0.15	−0.24	−0.42	−0.43	−0.43	−0.4	−0.01	−0.13	−0.05	−0.14	0.35	−0.2	0.12
	Kurtosis	−0.29	−0.4	−0.51	−0.42	−0.41	−0.33	−0.21	−0.59	−0.48	−0.73	−0.78	−0.46	−0.31	−0.52	−0.57

**p < 0.01, *N* = 614.

### Primary analysis

#### Measurement model

Structural equation modeling with a maximum likelihood estimation method was used to estimate the parameters (via AMOS Version 26). As the first step of testing the hypothesized structural model, the fitness of the measurement model was examined to confirm that all latent variables were acceptably represented by their parcels. In doing this, five latent factors were allowed to covary with no specified structural relations among them. All item parcels loaded significantly (*p* < 0.001) onto their respective factors, with standardized loadings ranging from 0.79 to 0.84 on outcome expectations, from 0.85 to 0.92 on academic engagement, from 0.76 to 0.83 on self-efficacy, from 0.76 to 0.79 on peer academic socialization and from 0.79 to 0.83 on parental academic socialization. Each of the fit indices suggested that the measurement model fits sample data very well (χ^2^/df = 2, RMSEA = 0.04 [90% CI = 0.031,0.049], CFI =0.97, TLI = 0.98 and SRMR = 0.027). Hence, the results of the measurement model supported that the indicators adequately measured their underlying latent factors.

#### Structural model

**Figure 3:**
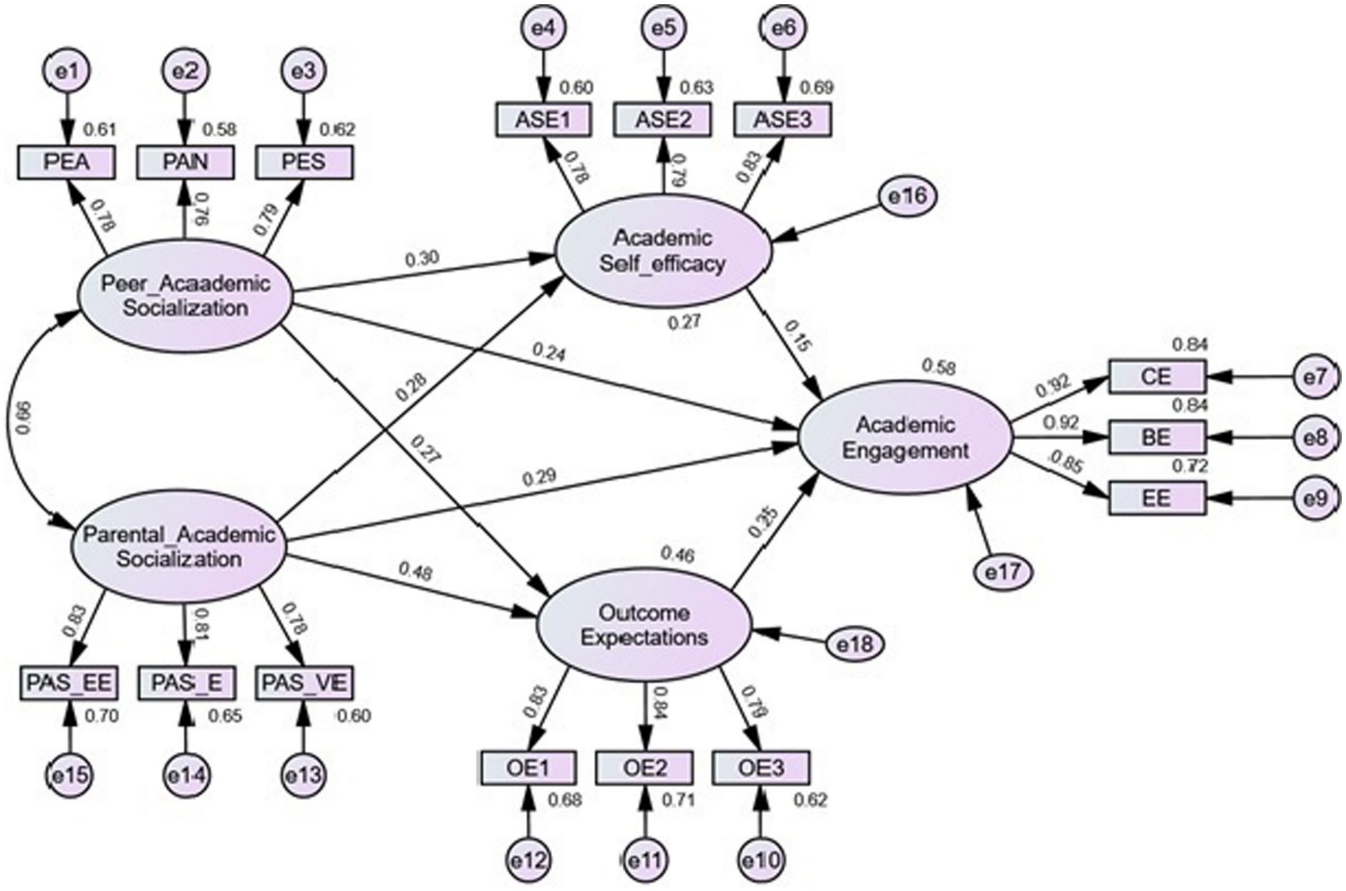
Tested structural model of academic socialization, motivational beliefs and academic engagement. PEA, Peer educational aspiration; PAN, peer academic norm; PES, peer effort socialization; PAS_EE, parental academic socialization -educational expectations; PAS_EE, parental academic socialization –effort; PAS_VE, parental academic socialization-value of education; CE, cognitive engagement; BE, behavioral engagement; EE, emotional engagement; ASE1, academic self-efficacy parcel 1; ASE2, academic self-efficacy parcel 2; ASE3, academic self-efficacy parcel 3; OE1, outcome expectations parcel 1; OE2, outcome expectations parcel 2; OE3, outcome expectations parcel 3. All *p* < 0.001.

As the measurement model fit the data well, the analysis proceeded to test the hypothesized structural model depicting the relations of the contextual and cognitive-person factors in explaining high school student academic engagement using the maximum likelihood estimation method. The result indicated an excellent model fit to the data (χ^2^/df = 2.1, CFI = 0.98, TLI = 98, RMSEA = 0.042 [90% CI = 0.033,0.051], and SRMR = 0.032). Also. the standardized residual correlation matrix supports the model as almost all values were within acceptable range. That is, the structural model fits the observed data well. All the hypothesized paths of the model were statistically significant, and they were in the predicted directions. The standardized direct effects are shown in Figure 3. Parental academic socialization was positively associated with students’ academic self-efficacy (*β* = 0.28, *p* < 0.001), outcome expectations (*β* = 0.48, *p* < 0.001), and academic engagement (*β* = 0.29, *p* < 0.001). Peer academic socialization was also positively associated with students’ academic self-efficacy (*β* = 0.30, *p* < 0.001), outcome expectations (*β* = 0.27, *p* < 0.001), and academic engagement (*β* = 0.24, *p* < 0.001). The cognitive-person factors, academic self-efficacy (*β* = 0.15, *p* < 0.001), and outcome expectations (*β* = 0.25, *p* < 0.001) were positively linked to academic engagement. Together, parental and peer socialization explained a significant amount of variance in academic self-efficacy (27.5%) and educational outcome expectations (46.4%). The four constructs in the model, as a whole, accounted for 58.4% of the variance in academic engagement of high school students.

#### Mediated effects

Bootstrap analysis, a more preferred method for testing mediation, was utilized to test the significance of indirect effects with the recommended 5,000 bootstrap samples (Fairchild and McQuillin, 2010; Collier, 2020) drawn with replacement. In this analysis, the indirect effects of peer academic socialization and parental academic socialization on student academic engagement through self-efficacy and outcome expectation were tested. The mediating effect is statistically significant if the confidence interval does not cross zero (Shrout and Bolger, 2002). Hence, the specific indirect effects of parental academic socialization on academic engagement of high school students through academic self-efficacy (*b* = 0.054, BC 95% CI = 0.02, 0.10, *p* < 0.001) and through outcome expectations (*b* = 0.156, BC 95% CI [0.090, 0.236], *p* < 0.001) were significant. Peer academic socialization had a significant indirect relation with academic engagement through self-efficacy (*b* = 0.059, BC 95% CI [0.026, 0.111], *p* < 0.001) and outcome expectations (*b* = 0.088, BC 95% CI [0.04, 0.155], *p* < 0.001). These findings indicate that cognitive-person variables partially mediated the relations between the social context and academic engagement of high school students.

## Discussion

The current study examined the structural relations among contextual (parental & peer academic socialization) and person-cognitive (self-efficacy and outcome expectations) factors in shaping the academic engagement of high school students in Ethiopia. As hypothesized, based on the integrative model of engagement and previous empirical evidence, the results showed that the posited model adequately fit the data, all paths were positive and significant, and the variables within the model accounted for a substantial amount of variance (58.4%) in the academic engagement of high school students. Also, the two exogenous factors of the model explained an adequate amount of variance in self-efficacy and outcome expectations. Parental academic socialization and peer academic socialization, together, accounted for 28% and 46% of the variance in self-efficacy and outcome expectations, respectively. Hence, the findings of the current study, from a sample of Ethiopian high school students, provided empirical support for the hypotheses posited in the development-in-sociocultural context model of engagement in learning (Wang et al., 2019, 2020).

### Role of parental academic socialization

As hypothesized, based on the integrative model of engagement and previous findings, high school students who perceived more positive academic socialization from parents endorsed greater self-efficacy to perform academic-related tasks and had stronger convictions that education would result in meaningful outcomes. Students who perceived parent-provided academic socialization messages as positive and encouraging had greater confidence in their abilities to perform academic tasks, associated positive outcomes with engagement in education, and were more likely engaged in academic affairs. Beyond being converged to the integrative model of engagement (Wang et al., 2020), these findings provide support for the perspective that parents are significant sources of self-efficacy and outcome expectations for children through social persuasion mechanisms (Bandura, 1997). When parents frequently convey messages to their children about high academic expectations, the value of education, and the importance of effort, they persuade their children that they are capable and expect positive outcomes in the future. In this way, parents bolster their children’s academic self-efficacy and positive outcome expectations for learning, which are considered the most important motivational beliefs in the academic domain. The finding that parental academic socialization significantly predicted academic self-efficacy corroborates the results of previous studies (e.g., Suizzo et al., 2016) and the conclusion that parents’ messages strongly influence children’s self-efficacy (Usher and Pajares, 2006). There was evidence that parental aspiration for their children’s postsecondary education, which was one aspect of parental academic socialization in this study, positively predicted students’ self-efficacy (Fan and Williams, 2010). Parental academic socialization was also related to high school students’ academic engagement both directly and indirectly through academic self-efficacy and outcome expectations. Hence, findings lead to the conclusion that parental academic socialization represented via three dimensions positively linked to students’ engagement in academic activities. The result was consistent with previous studies (Melby et al., 2008; Murray, 2009; Wilder, 2014) that suggested parental involvement in education matters for children’s engagement. Parents may socialize their children academically through multiple processes, such as the academic values they endorse, the expectations for academic success they set, and the experiences they share. The result of this study provides new evidence that parental involvement in the form of communicating beliefs about and expectations for their children’s success, linking education to future success, and stressing the reimbursement of exerting effort is an essential factor in fostering children’s academic engagement. The present finding suggests that academic socialization appears to be a fundamental substrate for student academic engagement. It has been reported that academic socialization has the strongest positive link with engagement during adolescence (Hill and Tyson, 2009; Wang et al., 2014). Fan and Williams (2010) also found that parental aspiration for their children’s postsecondary education, one aspect of parental academic socialization, positively predicted student engagement. As academic socialization is more likely to correspond with the developmental tasks of adolescents, such as achieving competence and accomplishment, its effect on high school student academic engagement seems to be acceptable.

Hence, the findings of the current study suggest that parental academic socialization matters for students to build higher academic self-efficacy, have positive outcome expectations for education, and be actively engaged in academic tasks. Students from parents who (a) recognized the utility of schooling, (b) had high expectations for their academic success, and (c) stressed the benefits of effort were likely to feel confident about their academic abilities, develop positive outcome expectations, and show a greater degree of academic engagement. It means that parents’ values for education, educational expectations, and beliefs in an effort which are conveyed and communicated through the process of academic socialization importantly determine their children’s motivational factors (i.e., self-efficacy, outcome expectations, and engagement) essential to achieve better academically.

### Role of peer academic socialization

In this study, high school students who perceived more positive academic socialization from peers endorsed greater self-efficacy to perform academic-related tasks and had stronger beliefs that education would result in important outcomes. Peer academic socialization was also related to high school students’ academic engagement both directly and indirectly through academic self-efficacy and outcome expectations. This finding supports that peers provide an important developmental context for adolescents (Furrer, 2010), and classmates and friends have a significant effect on students’ academic motivation and engagement (Wentzel, 2009). The effects of peer academic socialization observed in this study support the theories of peer group influence that have postulated peer effects may be conveyed directly through socialization processes that include peer pressure, modeling, reinforcement, and encouragement to follow group norms (Altermatt and Pomerantz, 2003; Lynch et al., 2013) as well as findings that members of adolescent peer groups are similar in many school-related adjustments such as in their learning motivation (Ryan, 2001), effortful behavior toward schoolwork, intrinsic value, interest, and enjoyment in schoolwork (Ryan, 2001; Shin and Ryan, 2014), and preference for challenge (Altermatt and Pomerantz, 2003). Therefore, the findings of this study are consistent with previous empirical evidence and provide support for the theoretical suppositions that peers have a greater potential to socialize the academic engagement of member students and basically, peers matter for individual student involvement in his/her education. Specifically, the finding that peer academic socialization predicts educational outcome expectations strengthens the thought that peers form a natural context for thinking about the future (Nurmi, 2004) and they are an essential source of future-related information for adolescents (Malmberg, 1996).

The findings that peer academic socialization shapes student engagement through multiple pathways suggest that peer groups have potential importance to the development of students’ engagement. The role peer academic socialization played in shaping student academic engagement could have different possible explanations. For example, peer group members: (a) share similar expectations about their future education (Kiuru et al., 2007), (b) are important sources of future-related information (Malmberg, 1996), (c) act as role models for each other in peer groups, (d) have a propensity for adhering to peer group norms and desire to fit in with those peers (Wang and Eccles, 2012), and (e) typically modify their levels of engagement to those of their peer group (Wang et al., 2018); and hence, all these are likely the mechanisms through which peers socialize individual student academic engagement. Together with previous evidence, the present study provides credence that peer context is an important substrate for the development of high school students’ engagement profiles. Students who belong to peer groups in which academic behaviors are normative are more likely to report a high level of engagement than students with less academically inclined peers.

### The roles of self-efficacy and outcome expectations as mediators

An integrative model of engagement has posited that self-appraisals or motivational beliefs are pathways through which a variety of external (e.g., academic socialization) assets influence learning engagement, and this was supported in the present study. The result supports the positive roles of self-efficacy and outcome expectations in predicting academic engagement, as students who have greater confidence in their abilities to do academic activities and stronger beliefs that being educated would lead to positive outcomes demonstrated a greater degree of academic engagement. These findings are consistent with the self-system motivational perspective, which posits that more positive self-beliefs are related to a greater level of engagement (Connell and Wellborn, 1991) and social cognitive theories that assert students with higher levels of academic self-efficacy and outcome expectations will engage in their learning more fully (Lent et al., 1994, 2000; Bandura, 1997). The positive relationship between academic self-efficacy and engagement also converges with evidence obtained in previous research (Linnenbrink and Pintrich, 2003; Martin and Rimm-Kaufman, 2015; Rimm-Kaufman et al., 2015; Liu et al., 2018; Navarro et al., 2019; Olivier et al., 2019). The relationship between outcome expectations and measures of academic engagement is consistent with the finding of Navarro et al. (2019) that found engineering outcome expectations positively predicted engineering academic engagement in undergraduate engineering students and Miller et al. (2021) who reported that students with higher math outcome expectations had a much higher likelihood of belonging to the ‘Moderately-to-Highly Engaged’ profile as compared to the ‘Minimally Engaged’ profile. Other studies also reported that task values are positively related to behavioral engagement (Fan, 2011; Wang and Eccles, 2013), intention to continue studying at school or beyond (Fan and Wolters, 2014), and positive classroom affect (Jiang et al., 2018) which support the present finding.

Self-efficacy and outcome expectations partially mediated the relations of academic socialization to student engagement. This result supports the integrative model that posits youth’s self-appraisals mediate the link between engagement and social context (Wang et al., 2020) and the social-cognitive perspective (Lent et al., 1994, 2000; Bandura, 1997) that states social environment operates through motivational self-beliefs to produce the required outcomes like academic engagement. According to the findings of this study, academic socialization facilitates academic engagement by enhancing self-efficacy and outcome expectations. With academic self-efficacy as a mediator of the relation between academic socialization and academic engagement, it was found that students whose parents and peers practice and forward encouraging socialization messages had higher academic self-efficacy, which in turn predicted better academic engagement. With the role of mediating the relation between academic socialization and academic engagement, outcome expectations of high school students whose parents and peers practice and convey positive academic socialization messages were higher, and it, in turn, predicted better academic engagement.

To sum up, the results revealed initial evidence for the utility of the integrated model of student engagement to predict the academic engagement of Ethiopian high school students who grew up and live within the collectivistic culture. Precisely, the findings that parental and peer academic socialization predicted academic engagement, both directly and indirectly through students’ academic self-efficacy and outcome expectations, suggest how family and peer contexts matter to foster student engagement in learning, particularly in the context where collectivism is more valued. These findings extend and support prior research on predicting student engagement from contextual and personal factors and shed light on how contextual and person-cognitive variables might contribute to the academic engagement of high school students. The result further suggests that self-efficacy and outcome expectations develop through comparable learning experiences, and they have comparable motivating effects to outcome variables (Lent et al., 1994; Bandura, 1997).

### Limitations and future research

Although this study has notable strengths, such as a large sample size, randomly drawn participants from different districts and schools, and being conducted in a country where almost all previous research on the issue has not been conducted, it has a number of limitations that should be considered while interpreting findings.

The first limitation is associated with the inclusion of variables. This study examined the influence of social systems on academic engagement by incorporating multiple aspects of family and peer contexts; however, there are other social agents (e.g., teachers) that could have been contributing factors to the outcome variable. Previous research has demonstrated that teachers’ messages strongly influence students’ self-efficacy (Usher and Pajares, 2006) and likewise may influence expected outcomes and, eventually, engagement in learning. The included psychological variables are also limited to self-efficacy and outcome expectations. Therefore, future research should consider teacher-related factors (e.g., psychological needs support, academic socialization) and other psychological constructs, especially those included in the integrative engagement model.

Second, the findings were based on cross-sectional design data. Hence, although (1) the proposed model was guided by previously developed theoretical frameworks and reported empirical findings, (2) SEM analysis provides important information about the possible direction of the relationships, and (3) results corroborate previous findings and theoretical predictions, cross-sectional study designs do not lead to firm conclusions regarding the causal ordering among the variables included. Third, the data was based on self-report measures. Self-perceptions are possibly best captured with self-reports, but for some constructs (e.g., academic engagement and parental academic socializations), data only from self-report measures may produce social desirability biases and narrow interpretations. Therefore, future researchers are encouraged to test the model using longitudinal designs and data from multiple sources to address these concerns. Fourth, it’s critical to remember that psychological study does not allow for the precise prediction of what will occur in a new setting based on past experiences. Hence, I encourage researchers to examine the model tested in this study in a way that addresses such concerns and see what the results would be. Finally, the other limitation of the current study may be associated with the influence of confounding variables and/or covariates. While prior studies have not explicitly suggested potential confounders to be considered in the context of the present study, there remains the possibility that unaccounted variables could impact the findings. Therefore, it is essential to interpret the findings while acknowledging these possibilities. Researchers interested in the predictors of student academic engagement should take this concern into account for future investigations. For instance, in the current study, it was assumed that there may not be significant variation in socioeconomic status (SES) within the target population to the extent that it would affect the outcome variable, hence SES was not included as a covariate in the model. However, future studies could benefit from the inclusion of status-based covariates, such as SES as highlighted by Wang et al. (2019), especially in populations where SES differences are more pronounced.

### Implications

This study, despite the limitations mentioned above, offers important contributions to theoretical understanding and practice regarding student academic engagement. The study demonstrated evidence for the theoretical model combining variables derived from contextual and personal domains, highlighting the role of person-cognitive factors as mechanisms explaining the relationship between contextual factors and student academic engagement. So, the findings provide substantial theoretical and empirical contributions to broaden knowledge on antecedents and the means of how they contribute to the academic engagement of high school students and suggest researchers in education and educational psychology pay more attention to the contextual and psychological constructs in the scholarship of student engagement. It would also provide support for the applicability of psychological constructs and models derived from theories fundamentally developed in Western cultures to Ethiopian culture. This is related to the issue of whether perspectives commonly supported in one culture (e.g., individualism) could be replicated among participants of other cultures (e.g., collectivism).

The current results, which come from participants with collectivistic cultural values and a different socio-economic background, make a significant contribution to the existing body of knowledge on student engagement which fundamentally represents a limited segment of the globe. They are particularly important in filling gaps in the understanding of student engagement across cultures as most of the previous research has been conducted in the Western contexts. This could be an attempt to address the concern in the field that concepts, practice, and empirical findings of psychology are mostly limited to Western countries but missing from African and other societies (Henrich et al., 2010; Berry, 2013).

Parallel with extending theoretical knowledge, the interrelations among constructs included in the present study suggest student academic engagement be increased and maintained through interventions targeting contextual and individual-level assets. According to the results, interventions targeting social contexts (e.g., academic socialization) may be associated with enrichments of all other variables. That is, promoting a positive academic socialization process appears to be a central component of intervention efforts in bolstering students’ self-efficacy, outcome expectations, and engagement in learning. Self-efficacy beliefs and outcome expectations are malleable enough and can be developed through targeted interventions. Interventions that focus on enhancing self-appraisals (e.g., self-efficacy and outcome expectancy), such as social persuasion and vicarious learning (Bandura, 1986), are helpful to improve student engagement in education, given that academic self-efficacy and outcome expectations are significantly related to student engagement. Furthermore, it would behoove parents, teachers, and school psychologists to focus on strengthening motivational beliefs as a way of enhancing academic engagement. Parents need to communicate to their children the positive outcomes of engaging in education, high academic expectations, and the consequence of lack of effort to promote positive self-efficacy beliefs and outcome expectations in the academic domain, which, in turn, craft motivated engagement in learning. School counselors or psychologists may design psychoeducational interventions that would bolster high school student’s academic efficacy and understanding of what they can gain from pursuing their education.

This study is also indicative of the need to pay due attention to how peer or friendship groups matter in determining the academic behaviors and success of children. The aspirations of others, academic norms, and beliefs in effort within adolescents’ peer and friendship groups were found to be ways by which peers may influence academic engagement. Adolescents tend to show similar levels of school-related beliefs, attitudes, and behaviors to others in their peer group, including school performance and academic self-perception (Ryan, 2001; Chen et al., 2003). Hence, as conformity to peer groups comes into prominence during adolescence, an intervention targeting peer and friendship groups would have a better effect in boosting students’ motivational beliefs and engagement in learning.

In conclusion, the findings were in line with theoretical predictions and earlier results and underscored the importance of parental and peer academic socialization for fostering students’ academic engagement. The substantial role of academic socialization and motivational beliefs (self-efficacy and outcome expectations) in explaining variance in academic engagement, which, in turn, leads to positive learning outcomes, suggests that educational policies and practices should be designed in a way that offers positive socialization experiences and boost motivational beliefs among students. The primary implication of the current findings is that interventions aimed at improving student self-efficacy, outcome expectations, and engagement, particularly for students who grew up and live within a collectivistic culture like Ethiopia, should take into account and integrate the most significant socializing agents in the academic domain (e.g., parents and peer groups). This study tends to encourage schools to plan and practice peer tutoring sessions, collaborative learning activities, and peer-based study groups to cultivate positive academic socialization among students.

The findings also offer an excellent opportunity for parents and teachers to be optimistic that they can cultivate children’s self-efficacy beliefs and outcome expectations as one means for fostering academic engagement, given that they have a strong desire for more active involvement of students in learning. Supported by the prior evidence that students with collectivistic value orientations benefit more from other or group-related sources such as vicarious experiences, and verbal and social persuasion as compared to students from countries with predominantly individualistic cultures (Klassen, 2004; Ahn et al., 2016), the present study suggests the need to paying due attention to social context in boosting Ethiopian students’ motivational beliefs and academic engagement.

## Data availability statement

The raw data supporting the conclusions of this article will be made available by the author, without undue reservation.

## Ethics statement

The studies involving humans were approved by the Ethics Committee of Institute of Teachers Education and Behavioral Science, Wollo University. The studies were conducted in accordance with the local legislation and institutional requirements. The participants provided their written informed consent to participate in this study.

## Author contributions

GW: Writing – original draft, Writing – review & editing.
