# Supplementary material for: Predictors of academic engagement of high school students: academic socialization and motivational beliefs
Source: Front Psychol. 2024 Jul 25;15:1347163. doi: 10.3389/fpsyg.2024.1347163 (PMC11310935; doi:10.3389/fpsyg.2024.1347163)
Supplement: Supplementary file 1 [file Data_Sheet_1.pdf]

Standardized Residual Covariances (Group number 1 - Default model)

	PAS_EE	PAS_E	PAS_VE	OE1	OE2	OE3	EE	BE	CE	ASE3	ASE2	ASE1	PES	PAN	PEA
**PAS_EE**	.00														
**PAS_E**	.29	.00													
**PAS_VE**	-.17	-.29	.00												
**OE1**	-.09	-.79	1.82	.00											
**OE2**	-.31	-.69	.75	.03	.00										
**OE3**	-.39	.76	.41	-.22	.16	.00									
**EE**	.48	.67	.60	.00	.15	-.11	-.05								
**BE**	.20	-.25	-.21	.24	-.34	-.39	-.25	-.06							
**CE**	-.67	.05	.39	-.13	-.58	-.24	-.07	.03	-.06						
**ASE3**	-1.11	-.70	-.46	-1.72	-2.31	-2.80	-.85	-1.00	-1.48	.00					
**ASE2**	.84	.96	.04	-1.60	-1.29	-1.35	.25	-.54	-1.06	.28	.00				
**ASE1**	.61	1.13	1.25	-.16	-.57	-1.25	1.25	1.99	.30	.05	-.44	.00			
**PES**	-.89	-.66	.58	1.22	-.11	.79	.63	-.44	-.80	-1.23	.06	.02	.00		
**PAN**	.27	.28	.57	-.23	-1.10	.00	.41	-.24	-.40	.47	.82	2.03	.16	.00	
**PEA**	.19	-.31	.26	.59	-.24	.06	2.01	.36	.23	-.60	.47	-.09	.05	-.30	.00

In the symmetric matrix displayed here, each residual covariance (see Residual Covariances), has been divided by an estimate of its standard error (Jöreskog & Sörbom, 1984). In sufficiently large samples, these *standardized residual covariances* have a standard normal distribution if the model is correct. So, if the model is correct, most of them should be less than two in absolute value.

.
